# Supra-second interval timing in bipolar disorder: examining the role of disorder sub-type, mood, and medication status

**DOI:** 10.21203/rs.3.rs-3006203/v1

**Published:** 2023-06-02

**Authors:** Victόria A. Müller Ewald, Nicholas T. Trapp, McCall E. Sarrett, Benjamin D. Pace, Linder Wendt, Jenny G. Richards, Ilisa K. Gala, Jacob N. Miller, Jan R. Wessel, Vincent A. Magnotta, John A. Wemmie, Aaron D. Boes, Krystal L. Parker

**Affiliations:** a Department of Psychiatry, The University of Iowa, Iowa City, Iowa, United States of America; b Department of Pediatrics, The University of Iowa, Iowa City, Iowa, United States of America; c Department of Psychological & Brain sciences, The University of Iowa, Iowa City, Iowa, United States of America; d Department of Neurology, The University of Iowa, Iowa City, Iowa, United States of America; e Department of Molecular Physiology and Biophysics, The University of Iowa, Iowa City, Iowa, United States of America; f Department of Neurosurgery, The University of Iowa, Iowa City, Iowa, United States of America; g Department of Radiology, The University of Iowa, Iowa City, Iowa, United States of America; h Iowa Neuroscience institute, The University of Iowa, Iowa City, Iowa, United States of America; i Institute for Clinical and Translational Science, The University of Iowa, Iowa City, Iowa, United States of America; j Department of Psychological and Brain sciences, Villanova University, Villanova, Pennsylvania, United States of America; k St. Luke’s Hospital, Cedar Rapids, Iowa, United States of America

**Keywords:** Cognition, Bipolar disorder, Medication status, Depression, Antipsychotic

## Abstract

**Background:**

Widely reported by bipolar disorder (BD) patients, cognitive symptoms, including deficits in executive function, memory, attention, and timing are under-studied. Work suggests that individuals with BD show impairments in interval timing tasks, including supra-second, sub-second, and implicit motor timing compared to the neuronormative population. However, how time perception differs *within* individuals with BD based on BD sub-type (BDI vs II), mood, or antipsychotic medication-use has not been thoroughly investigated. The present work administered a supra-second interval timing task concurrent with electroencephalography (EEG) to patients with BD and a neuronormative comparison group. As this task is known to elicit frontal theta oscillations, signal from the frontal (Fz) lead was analyzed at rest and during the task.

**Results:**

Results suggest that individuals with BD show impairments in supra-second interval timing and reduced frontal theta power compared during the task to neuronormative controls. However, within BD sub-groups, neither time perception nor frontal theta differed in accordance with BD sub-type, mood, or antipsychotic medication use.

**Conclusions:**

This work suggests that BD sub-type, mood status or antipsychotic medication use does not alter timing profile or frontal theta activity. Together with previous work, these findings point to timing impairments in BD patients across a wide range of modalities and durations indicating that an altered ability to assess the passage of time may be a fundamental cognitive abnormality in BD.

## Background

Current treatments for bipolar disorder (BD) largely focus on mood symptoms (1–4). However, changes in cognitive functioning, including deficits in memory, executive function, attention, planning, and timing (5–8), are common and may even precede a formal BD diagnosis (9). Cognitive symptoms are reported by patients with bipolar I disorder (BDI) and bipolar II disorder (BDII), and are present even in a euthymic state (10). As these symptoms are widely reported and linked to lowered quality of life (11), studies of cognitive symptoms within BD are imperative to a comprehensive understanding of the disorder.

Previous work has identified timing deficits as a cognitive abnormality consistently presented by BD patients (12). Using finger tapping, auditory temporal bisection, and single-cue delay eye blink conditioning, Bolbecker and colleagues showed sub-second interval timing impairments in BD (13–15). Using time production and estimation tasks, Bschor and colleagues showed supra-second interval timing deficits in BD ranging from durations of 7 to 109 seconds. Interval timing depends on diffuse neural networks including the cerebello-thalamo-cortical network and the cortico-striatal network (16, 17). In BD, abnormalities in nodes of these networks have been previously reported, including frontal cortex, thalamus, and cerebellum (18–22), providing a mechanistic explanation for the wide-ranging deficits in timing observed in the disorder. Previous work suggests that impairments in interval timing correlate with abnormal fronto-central theta oscillations in patients with SCZ (23, 24). Substantial genetic and symptomatic overlaps between SCZ and BD have been suggested (4, 25). However, it is unclear if the deficits in timing and frontal oscillations observed in SCZ extend to BD.

While extensive work has compared time processing between individuals with BD and other clinical populations or neuronormative controls, there is a paucity of work examining which *specific* characteristics within BD are linked to timing deficits. The relationship between depressive symptoms and time-perception slowing is well established and referred to as depressive time dilation (26). Additionally, work suggests that manic patients also show alterations in time processing, although works differ in effect directionality (12, 27). However, is unknown if the timing deficits observed in BD are linked to mood status at the time of assessment, or if they are a stable characteristic present even in the absence of mood symptoms.

Additionally, while some reports suggest that cognitive impairments in BD can improve in conjunction with mood-symptom treatment (28), other reports suggest that cognitive symptoms may worsen in conjunction with mood treatment (25, 28). It has been suggested that antipsychotic medications may impair measures of general intellectual functioning, working memory, and cognitive set-shifting (6), likely due to reductions in information processing speed. Indeed, although the relationship between different medication types, including antidepressants and stimulants, and cognitive functioning in BD have been studied, antipsychotic-use is the only medication related variable with consistently significant impacts on cognition (6).

Finally, although it is established that individuals with BD show impairments in timing, work has not explored how this may differ between bipolar disorder sub-types. Given differences in cycling speed and manic episode strength between disorder sub-types (29), differences in time perception could be expected. However, work also suggests similar cognitive profiles between BDI and BDII (10), adding a layer of complexity to this debate.

To address these questions, we administered a supra-second interval timing task (ITT) to participants with BD and neuronormative controls (CT), while simultaneously recording electroencephalographic (EEG) activity. We hypothesized that individuals with BD would show impaired supra-second ITT performance, in agreement with previous work. Because of previous work with SCZ patients, we further hypothesized that BD patients would show reduced frontal theta power compared to the CT group during the ITT. Finally, we assessed differences in ITT performance and frontal theta in BD depending on disorder sub-type, mood, or antipsychotic medication status.

## Methods

### Subjects

Twenty-four participants (20 females, 4 males) with a DSM-IV diagnosis of BDI (16 subjects) or BDII (8 subjects) were recruited from the Iowa Longitudinal Database (Tables 1 and 2). Subjects had diagnoses confirmed by a board-certified psychiatrist at the University of Iowa Hospitals & Clinics. Medication status was stable for a minimum of 30 days prior to enrollment and was not altered for the present study (Table 3). Individuals who reported illicit drug use within 6 months of study commencement were excluded from participation. Mood was assessed via the Montgomery-Asberg depression rating scale (30). A depressed state was defined as a score greater than 10 (31). Six CT subjects were included as a neuronormative comparison group. CT subjects did not have a history of neuropsychiatric disorders.

In accordance with federal and institutional guidelines, all procedures including informed consent were approved by the University of Iowa Institutional Review Board and are in accordance with the Declaration of Helsinki.

### Tasks

#### Interval timing task

Concurrent with EEG acquisition, participants performed a supra-second ITT. Participants completed the task sitting in front of a Dell 20” monitor with a 60 Hz refresh rate and 4096×2304 screen resolution. White times new roman size 40 text appeared on a black background in the middle of the screen. Participants received verbal instructions on how to perform the task from the experimenter and read the same set of instructions on the computer screen. All participants were instructed not to count time in their head. To start each trial, a number indicating the interval to be estimated by the participant (“3” for the short interval/SIT or “12” for the long interval/LIT) appeared on the screen. Participants pressed the space bar to start the trial and to indicate their judgement of the elapsed interval, thus ending the trial. Response accuracy feedback was given for every trial immediately following the button press. The experiment consisted of a total of 80 trials (40 SIT trials & 40 LIT trials) presented in pseudo-random order.

#### Resting state

Resting-state recordings were conducted before the ITT task, lasting 5 minutes. Participants sat in a chair, were instructed to keep their eyes open, look forward, and let their mind wander.

### Electroencephalography (EEG)

#### EEG acquisition

A BrainVision 64-channel active electrode system with Ag/AgCl electrodes was used to collect EEG (Morrisville, NC). A custom-made electrode cap was utilized, which included electrode placements that are not typical of the International 10–20 system (32). Electrodes PO3 and PO4 were substituted by electrodes I1 and I2 which flanked the Iz electrode. These were situated at the back of the head over the inion and were not analyzed as a part of the present experiment. At the beginning of the recordings, impedances were reduced using high viscosity electrode gel for active electrodes (EASYCAP, Munich, Germany). Impedance for all electrodes was kept at or below 15 kΩ for the duration of the recording. Data were acquired at 500 Hz and referenced online to Pz.

#### EEG analyses

Analyses focused on electrode Fz, as frontal theta oscillations are typically maximal at this site (33). Frequency bands were defined as: delta (1–4 Hz), theta (4–8 Hz), alpha (8–13 Hz), beta (13–30 Hz), and gamma (30–50 Hz).

Data were preprocessed using custom MATLAB (MathWorks, Natick, MA) scripts based on EEGLAB (34) functions. Data were sequentially high-pass filtered at 1Hz, and low-pass filtered at 50Hz, the transition bandwidth was set to twice the cutoff frequency (−6 dB) for cutoff <= 1Hz and 25% cutoff frequency for cutoff > 8Hz. Trials containing nonstereotypic artifacts were removed manually, resulting in exclusion of 18% of trials on average. Continuous data were rereferenced offline to average voltage. Eyeblinks and saccades were removed using independent component analysis.

Data were epoched as follows: for the interval timing task, data were epoched around the presentation of the timing cue. For the SIT, the epoch ranged from 1 second before cue presentation to 5 seconds after cue presentation. For the LIT, data were epoched from 1 second before cue presentation to 15 seconds after cue presentation. For resting-state analyses, continuous data were epoched into 20s intervals to maintain epochs at approximately the same size between the two tasks. Quantification of band power was conducted using the fast-Fourier transform method. Relative power at each frequency band was defined as the proportion of the overall spectral power distribution occupied by each frequency band, quantified using the MATLAB function trapz.

### Statistical analyses

#### Participant characteristics

Demographic characteristics were compared between individuals in the BDI, BDII, and CT groups using a Pearson’s Chi Square for categorical variables and a one-way ANOVA for continuous variables. Categorical variables were: sex, race, education and handedness. Age was the only continuous variable. Additionally, propensity scores were generated to assess whether age, sex, race, and years of education were associated with the probability of a participant being in the BD group vs. the CT group.

#### Interval timing task performance and band power

To assess performance on the ITT, participants’ time estimates for the SIT/LIT intervals were fit with Gaussian distributions using custom-written MATLAB routines. Timing accuracy and precision were estimated by calculating peak time and CV measures, respectively. The peak time index represents the accuracy of participants’ responses and was calculated using the best fit estimate of the Gaussian distribution. The CV index represents the precision of participants’ responses and was calculated by dividing the response standard deviation by peak time. T-tests were conducted in GraphPad Prism (San Diego, California) to statistically assess performance differences between groups.

Statistical comparisons of power at each oscillation band were compared between groups in GraphPad Prism using t-tests. Multiple comparisons were corrected for using Tukey’s multiple comparisons test.

Statistical outliers were defined as individuals with scores 2 standard deviations above/below their group mean and excluded from the analysis. Mean, standard error of the mean (SEM), and number of outliers excluded for each group are expressed as [GROUP NAME mean ± SEM (number of outliers excluded)].

## Results

### Participant characteristics

**Figure 1.**
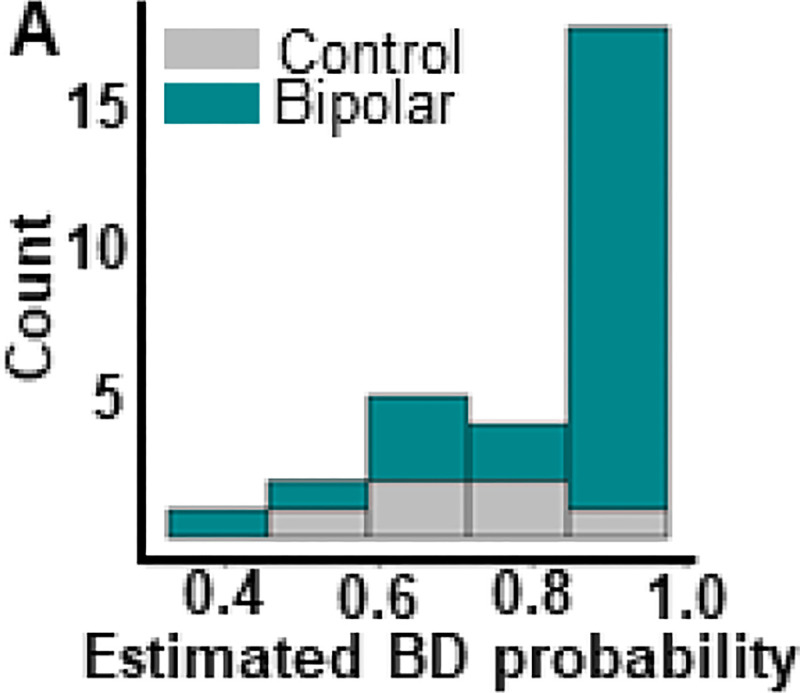
Propensity histogram for neuronormative control and bipolar patient groups. Distribution of control (grey) and bipolar (teal) propensity scores along X axis indicates similar distribution of demographic variables for both groups. Nearly all participants have an estimated probability of being in the BD group that is greater than 0.5 because the majority of our sample belongs to the BD group. This graph indicates that while our groups may not be perfectly balanced, the degree of imbalance between them is not indicative of significant sampling bias.

The demographic characteristics age, sex, race education and handedness did not significantly differ between individuals in the BDI, BDII, and CT groups (Table 1). Additionally, although the study population was heavily skewed towards the BD group, propensity scores did not provide strong evidence that participants in the BD vs. the CT groups substantially differed with regards to their age, race, sex, and years of education (Figure 1).

### Comparison between BD and CT groups

**Figure 2.**
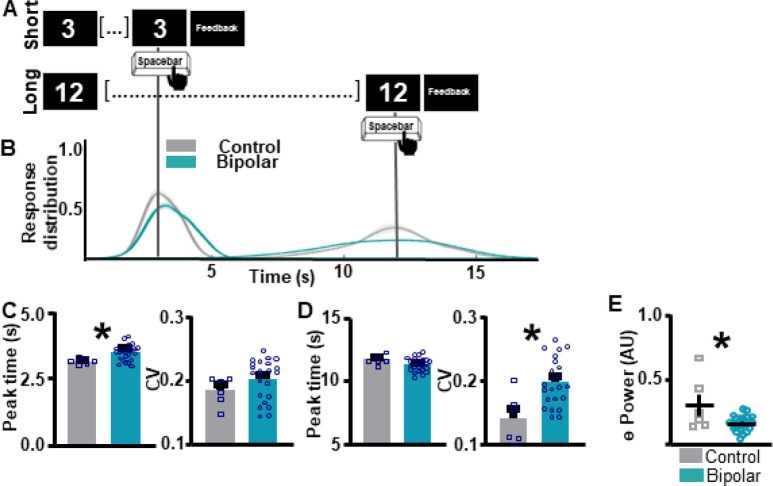
Individuals with bipolar disorder show impairments in supra-second interval timing and abnormal frontal theta compared to neuronormative controls. **A.** Schematic diagram of supra-second interval timing task. Trials begin when participants are shown a 3s or a 12s timing cue. Participants press the spacebar to indicate their estimation of the target interval. **B.** Response distribution for neuronormative controls vs. individuals with bipolar disorder. **C.** Individuals with bipolar disorder over-estimate the short interval compared to controls **[left]**. No differences in response distribution were detected **[right]**. **D.** Individuals with bipolar disorder do not differ from controls in estimation of the long interval duration **[left]**, however, individuals with bipolar disorder have a significantly wider response distribution compared to controls **[right]**. **E.** Individuals with bipolar disorder show lower theta power compared to individuals in the neuronormative control group during the supra-second interval timing task. Mean and standard error of the mean plotted in bar graphs. Dots represent values from individual subjects. * p < 0.05

Individuals with BD show impaired supra-second ITT performance compared to the CT group (Figure 2A–B). For SIT, individuals with BD show an over-estimation of the target duration compared to the CT group, as quantified by the peak time index (*t*_(27)_ = 2.61, *p* = 0.0146 [BD 3.45 ± 0.0616 (1); CT 3.17 ± 0.0.0466 (0)]; Figure 2C [left]). Response distribution, quantified by the CV index, did not differ between groups (*t*_(27)_ = 1.33, *p* = 0.192 [BD 0.2032 ± 0.00655 (1); CT 0.184 ± 0.00883 (0)]; Figure 2C [right]). For the LIT, peak response times did not differ between BD and CT groups (*t*_(28)_ = 1.576, *p* = 0.1262 [BD 11.36 ± 0.1084 (0); CT 11.73 ± 0.1620 (0)]; Figure 2D[left]). However, individuals with BD showed significantly lower CV indices, indicating higher variability in response times compared to the CT group (*t*_(27)_ = 3.345, *p* = 0.0024 [BD 0.198 ± 0.00778 (1); CT 0.1406 ± 0.161 (0)]; Figure 2D[right]).

**Figure 3.**
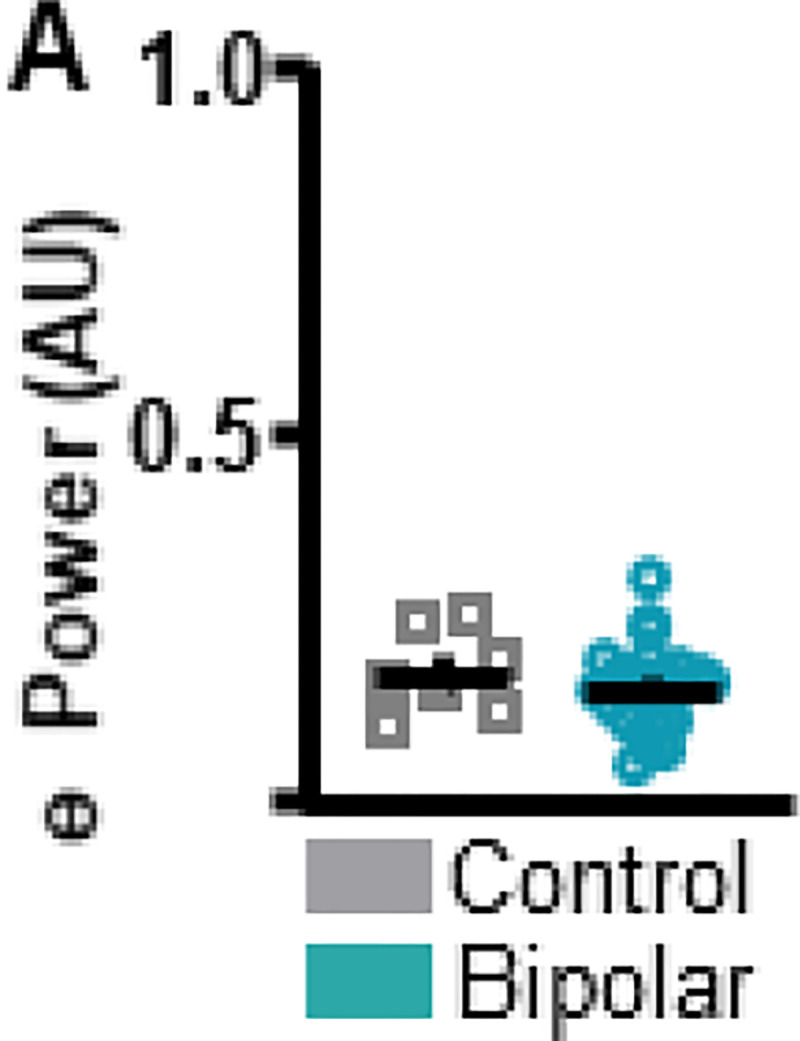
Individuals with bipolar disorder and neuronormative controls do not differ in frontal theta power at rest. **A.** To assess resting-state differences in theta power between bipolar disorder and neuronormative control groups resting-state data were analyzed. No differences in resting-state theta power were identified between neuronormative control and bipolar groups. Mean and standard error of the mean plotted in bar graphs. Dots represent values from individual subjects. * p < 0.05

During the ITT, individuals with BD showed lower frontal theta power compared to the CT group (*t*_(27)_ = 2.992, *p* = 0.0059 [BD 0.159 ± 0.0116 (1); CT 0.3000 ± 0.0842 (0)]; Figure 2E). No differences in power were detected between the BD and the CT groups during the ITT for any other frequency bands (**Supplemental Figure 1**). Although theta power values of a single CT subject are markedly higher than the remainder of the CT subjects, this data point was not excluded, as it does not fit the statistical outlier criteria as described in the methods section. To assess if differences in theta power between BD and CT groups were task-specific, resting-state data were analyzed (Figure 3). There were no significant differences in theta power between BD and CT groups (*t*_(30)_ = 0.8343, *p* = 0.4107 [BD 0.147 ± 0.009901 (1); CT 0.248 ± 0.0488 (1)]) during rest.

### Comparisons within BD sub-groups

**Figure 4.**
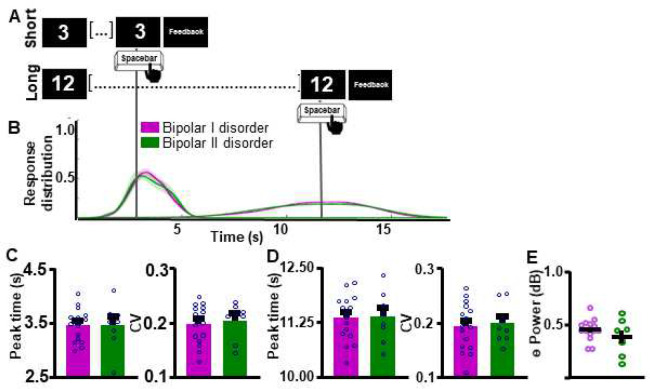
Interval timing performance and frontal theta power do not differ as a function of bipolar disorder sub-type. A. Schematic diagram of supra-second interval timing task. Trials begin when participants are shown a 3s or a 12s timing cue. Participants press a button to indicate their estimation of the target interval. **B.** Response distribution for individuals with bipolar I or bipolar II disorder. **C-D.** Groups do not differ in time estimation for the short **[C]** or the long **[D]** intervals. **E.** Frontal theta power during the ITT did not differ between groups. Mean and standard error of the mean plotted in bar graphs. Dots represent values from individual subjects. * p < 0.05

Within our group of individuals with BD, we first assessed if BD disorder sub-type differentially affected supra-second interval timing ability and associated frontal theta power. Response curves suggest that individuals with BDI and BDII did not differ in their supra-second ITT performance (Figure 4A–B). Peak time and CV indices did not differ between groups for either the SIT (Peak time: *t*_(22)_ = 0.02449, *p* = 0.9807 [BDI 3.45 ± 0.0760 (0); BDII 3.45 ± 0.153 (0)]; Figure 4C [left]; CV: *t*_(22)_ = 0.4230, *p* = 0.6764 [BDI 0.198 ± 0.00879 (0); BDII 0.204 ± 0.0120 (0)]; Figure 4C [right]) or the LIT (Peak time: *t*_(22)_ = 0.9181, *p* = 0.9277 [BDI 11.35 ± 0.130 (0); BDII 11.37 ± 0.206 (0)]; Figure 4D [left]; CV: *t*_(22)_ = 0.3181, *p* = 0.7534 [BDI 0.192 ± 0.0109 (0); BDII 0.195 ± 0.0130 (0)]; **Supplemental Figure 4D [right]**) intervals. Additionally, theta power during the ITT did not differ between individuals with BDI vs. BDII (*t*_(21)_ = 1.268, *p* = 0.2188, one BD outlier excluded, Figure 4E).

**Figure 5.**
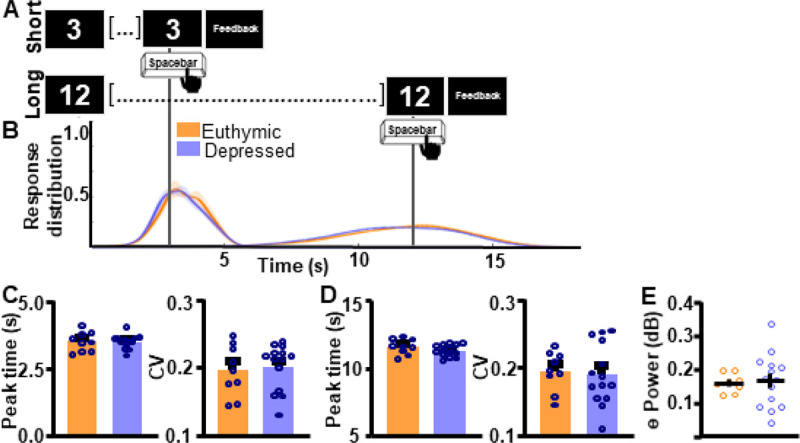
Interval timing performance and frontal theta power do not differ as a function of mood. **A.** To assess task-wide differences in oscillatory activity data from the whole interval-timing task were analyzed. **B.** Response distribution for individuals with bipolar disorder who were either euthymic or depressed at the time of data collection. **C-D.** Groups do not differ in time estimation for the short **[C]** or the long **[D]** intervals. **E.** Frontal theta power during the ITT did not differ between groups. * p < 0.05

Next, we assessed if ITT performance and associated frontal theta differed by mood status (i.e. depressed vs. euthymic) within the BD group. Response curves suggest that supra-second ITT performance does not differ between depressed vs. euthymic individuals (Figure 5A–B). Peak time and CV indices did not differ between groups for the SIT (Peak time: *t*_(20)_ = 0.05827, *p* = 0.9541 [BD 3.502 ± 0.0725 (0); CT 3.510 ± 0.121 (0)]; CV: *t*_(21)_ = 0.3629, *p* = 0.7203 [BD 0.2011 ± 0.00900 (1); CT 0.195 ± 0.0124 (0)]; Figure 5C) or the LIT (Peak time: *t*_(21)_ = 1.333, *p* = 0.1969 [BD 11.30 ± 0.116 (0); CT 11.57 ± 0.186 (0)]; CV: *t*_(21)_ = 0.3012, *p* = 0.7662 [BD 0.189 ± 0.0120 (0); CT 0.194 ± 0.00967 (0)]; Figure 5D) intervals. Frontal theta power also did not differ between groups as shown in Figure 5E (*t*_(20)_ = 0.1963, *p* = 0.8463 [BD 0.165 ± 0.0216 (1); CT 0.160 ± 0.00999 (0)]).

**Figure 6.**
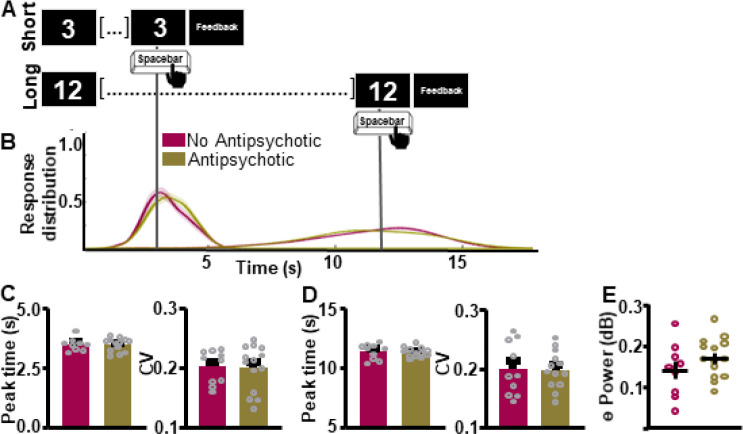
Interval timing performance and frontal theta power do not differ as a function of antipsychotic medication status. **A.** Response distribution for individuals with bipolar disorder divided by anti-psychotic medication status. **B-D.** Groups do not differ in time estimation for the short **[C]** or the long **[D]** intervals. **E.** Frontal theta power during the ITT did not differ between groups. Mean and standard error of the mean plotted in bar graphs. Dots represent values from individual subjects. * p < 0.05

Finally, we assessed if ITT performance and associated frontal theta differed by antipsychotic medication-use within the BD group. Response curves suggest that supra-second ITT performance was not significantly associated with differences in antipsychotic medication status (Figure 6A–B). Peak time and CV indices did not differ between groups for the SIT (Peak time: *t*_(20)_ = 0.1367, *p* = 0.8927 [BD 3.46 ± 0.0780 (0); CT 3.47 ± 0.0900 (0)]; CV: *t*_(22)_ = 0.1525, *p* = 0.8802 [BD 0.199 ± 0.0103 (0); CT 0.201 ± 0.00898 (0)]; Figure 6C) or the LIT (Peak time: *t*_(21)_ = 0.2647, *p* = 0.7938 [BD 11.29 ± 0.118 (1); CT 11.35 ± 0.198 (0)]; CV: *t*_(21)_ = 0.02555, *p* = 0.9799 [BD 0.198 ± 0.00886 (1); CT 0.1987 ± 0.01428 (0)], Figure 6D) intervals. Frontal theta power during the ITT also did not differ between groups Figure 6E (*t*_(21)_ = 1.284, *p* = 0.2133 [BD 0.171 ± 0.0130 (1); CT 0.140 ± 0.0220 (0)]).

## Discussion

The objective of the present work was to assess supra-second ITT performance in individuals with BD. Specifically, we were interested in whether BD disorder sub-type, mood, or antipsychotic medication-use altered supra-second interval timing in our cohort of patients. Our results suggest that, although ITT performance and frontal theta were decreased in the BD group compared to the CT group, within BD sub-groups there were no differences in ITT performance or frontal theta power. Together with previous work indicating that individuals with BD show impairments in supra-second (12, 27), sub-second (13, 15), and implicit motor timing (14), our work suggest that an altered ability to assess the passage of time may be a fundamental cognitive abnormality in BD.

### Trait vs. state abnormalities in BD

Although cognitive impairments are widely reported by BD patients (10), few reports have attempted to triangulate which cognitive impairments may be state vs. trait characteristics of BD. Because clinical characteristics vary between BDI and BDII, and because previous work suggests a link between depressed mood/antipsychotic medication-use and altered temporal processing, we assessed if these characteristics varied within our group of BD patients.

First, our results indicate that supra-second timing performance is not altered as a function of BD disorder sub-type. The extent to which cognitive profiles differ between BDI and BDII is debated in the literature. While some work suggests that BDI presents with more significant cognitive impairments (8), other studies suggest similar levels of cognitive impairments between the two sub-groups (10). Indeed, recent work suggests that BDII patients show impaired performance in neuropsychological tests including attention/working memory, executive function, verbal and visual memory, and motor speed compared to neuronormative controls (35). Our results add to this body of literature, indicating that cognitive impairments in the supra-second interval timing domain, do not differ by BD disorder sub-type.

Additionally, our work suggests supra-second interval timing abilities do not differ between depressed and euthymic BD patients. The lack of distinction between these two groups is interesting given the well-established link between depression and a slowing of time perception in the supra-second domain (36). However, previous work suggests that 40–60% of euthymic BD patients may present with some sort of neurocognitive impairment (8). Indeed, work by Martino and colleagues (37) assessed six cognitive domains (attention, verbal memory, language, psychomotor speed, executive function, and facial emotional recognition) in BD and found that 62% of euthymic BD patients showed cognitive impairments, with 40% of patients showing 1 or 2 impaired domains, and 22% of patients showing impairments in 3 to 5 domains. Our findings thus suggest an additional domain – supra-second interval timing - where BD patients show impairments even when in the euthymic state, adding to the growing literature indicating that cognitive markers are fundamental characteristics of the disorder.

Finally, our work suggests that antipsychotic medication-use does not alter supra-second timing in BD patients. Past work suggests a negative association between antipsychotic medication-use and IQ in BD (38). Specifically, tests of working memory, set-shifting, and response initiation/inhibition are negatively affected by antipsychotic medication-use (6). However, not all cognitive measures in BD are affected by antipsychotic medication-use, including measures of response planning and general working memory. In the context of this literature, our negative findings concerning antipsychotic use and interval timing are surprising. However, the dependence of timing abilities on working memory and response planning, two cognitive features not altered by antipsychotic use, could explain these findings. Another possibility is that participants were on low antipsychotic doses. Because antipsychotic dose is related to the degree of cognitive impairment, this could explain the lack of group difference. However, dose information was not collected, thus this analysis cannot be conducted leaving space for future work.

### Frontal theta during the ITT

The present work identified abnormalities in frontal (Fz) theta oscillations during the ITT in BD patients compared to CT participants. Previous work suggests that ITT performance increases frontal theta power compared to rest. This pattern of activity was detected for the CT group, where visual inspection of ITT vs. resting state graphs suggests that frontal theta power was higher during the ITT (Figures 2E vs. 3A). However, this pattern was not detected for the BD group, where average power stayed approximately the same during task and rest. This suggests a failure in the mechanisms subserving time perception in the BD group, expressed electrophysiologically as unaltered frontal theta power and behaviorally as impaired supra-second interval timing.

Previous work suggests that, compared to neuronormative controls, patients with SCZ show abnormal frontal low frequency (delta + theta) activity during the ITT (23). Our work suggests that the relationship between abnormal frontal theta and impaired ITT performance may not be a characteristic of SCZ specifically, extending to BD as well. If this characteristic extends outside of the schizoaffective domain, however, remains to be determined.

Finally, in SCZ patients, work suggest that abnormalities in theta power in the 500ms window following timing-cue presentation is related to abnormal supra-second ITT performance (23). Although the primary objective of the present work was to analyze ITT performance and theta power *within* BD sub-groups, not between BD and CT groups, because of this previous SCZ work, secondary analyses were added to identify specific epochs of altered theta power during the task. These analyses were time-locked to cue presentation and response. Results suggest that oscillatory abnormalities in BD were not time-locked to the post-cue interval as they were in SCZ (Supplemental Figures 2B-C and 3B-C). One surprising finding from the present dataset is that individuals with BD showed lower theta power surrounding the response for the long interval (Supplemental Figure 3F), but not the short interval (Supplemental Figure 2F). This parallels performance data where BD patients show altered precision estimates for the long interval (Figure 2D [right]), but not the short interval (Figure 2C [right]). These results could indicate that frontal theta power is more closely linked with precision than response accuracy. However, further work is necessary to substantiate this claim.

### Pathophysiology of bipolar disorder

Using timing task performance to triangulate *single regions* which may be abnormal in BD presents a challenge, as the neuroanatomy of time processing is famously diffuse (17) involving the coordinated functioning of multiple brain regions and neurotransmitter systems. One mechanism underlying the altered ITT performance observed in the present work may be the abnormal functioning of the dopamine system in individuals with BD. Indeed, the dopamine hypothesis of BD, which proposes intrinsic dysregulation of dopamine receptor transporter homeostasis (3, 39), is widely used to explain the pathophysiology of this disorder. Additionally, in other disorders where dopaminergic pathway function is altered, such as SCZ, Parkinson’s, or Huntington’s disease, abnormalities in temporal processing have also been reported (17).

However, the absence of an effect of antipsychotic treatment on ITT performance weighs against the interpretation of timing deficits being caused by dopaminergic system abnormalities, as this medication class primarily targets the dopamine system.

Another possible mechanism subserving the ITT performance and frontal theta deficits identified in the present work is the well-characterized frontal cortical abnormalities observed in individuals with BD including reductions in cortical grey matter (18, 40). Indeed, compromised frontal cortical activity has been linked to abnormalities in supra-second interval timing (17). This suggests a suggests a failure in the frontal mechanisms subserving time perception in BD patients, expressed electrophysiologically as unaltered frontal theta power and behaviorally as impaired supra-second interval timing.

### Limitations

The present sample is skewed towards BD patients, as the CT group comprises 20% (n = 6) of the total study population while the BD group comprises 80% (n = 24) of the population. Because of this, the precision of estimates where comparisons between CT and BD groups are made are limited. However, one indicator of the reliability of these findings, is that they are in agreement with previous work which has identified differences in supra-second ITT performance between CT and BD groups (12, 27).

Finally, we were unable to assess how mania alters supra-second interval timing performance, as none of our BD patients were in a manic state. This question is of particular interest as results are not consistent within the literature: while some studies suggest that manic patients under-estimate supra-second intervals (12), other suggests that manic patients over-estimate such intervals (27). However, this remains an open question for future work.

## Conclusions

Although previous work has established timing deficits in BD, it is unclear if these cognitive abnormalities are due to secondary characteristics associated with BD, such as medication and mood, or if they are a fundamental characteristic of the disorder. In this study, we assessed whether BD sub-type (BDI vs. BDII), mood, or antipsychotic medication-use differentially affected BD patients’ ITT performance and associated frontal theta. Results suggest that ITT performance and frontal theta do not differ between BD sub-types, mood, or antipsychotic medication status. Together with previous work assessing interval timing in BD, these results suggest that an altered ability to assess the passage of time may be a trait cognitive abnormality in BD.

## Figures and Tables

**TABLE 1.** Participant Demographics

Baseline Characteristic	Controls (n=6)	Bipolar type I (n=16)	Bipolar type II (n=8)	p-value

Age				0.948
Mean (SD)	37.2 (10.3)	37.2 (13.5)	35.5 (12.1)	
Sex				0.212
Female	3 (50%)	13 (81.25%)	7 (87.50%)	
Male	3 (50%)	3 (18.75%)	1 (12.50%)	
Race				0.471
White	4 (66.67%)	12 (75%)	6 (75%)	
Black/African American	0 (0%)	3 (18.75%)	0 (0%)	
Asian	1 (16.67%)	0 (0%)	1 (12.50%)	
Other	1 (16.67%)	1 (6.25%)	1 (12.50%)	
Education				0.138
No High School	0 (0%)	2 (12.50%)	0 (0%)	
High School	0 (0%)	5 (31.25%)	3 (37.50%)	
Associate’s/Bachelor’s	3 (50%)	7 (43.75%)	5 (62.50%)	
Post-Graduate	3 (50%)	2 (12.50%)	0 (0%)	
Handedness				0.126
Right	5 (83.33%)	16 (100%)	8 (100%)	
Left	1 (16.67%)	0 (0%)	0 (0%)	

A one-way ANOVA was used to assess differences in continuous variables, while a Chi-square analysis was used to assess differences between categorical variables.

**TABLE 2.** Comorbidities reported at enrollment for bipolar group

Comorbidity	n (%)
Generalized Anxiety Disorder	5 (20.8%)
Post-Traumatic Stress Disorder	4 (16.67%)
Panic Disorder	2 (8.33%)
Borderline Personality Disorder	2 (8.33%)
Attention-Deficit/Hyperactivity Disorder	2 (8.33%)
Migraine	1 (4.17%)
Fibromyalgia	1 (4.17%)
Other Medical Condition	5 (20.8%)

**TABLE 3.** Medications reported at enrollment for bipolar group

Type of Medication	n (%)
Any antidepressant	18 (75%)
SSRI	4 (16.7%)
SNRI	6 (25%)
Atypical antidepressant	11 (45.8%)
Atypical antipsychotic	14 (58.3%)
Lithium	6 (25%)
Benzodiazepine	10 (41.67%)
Stimulant	4 (16.7%)
Anticonvulsant	11 (45.8%)
Opioid	2 (8.3%)

SSRI, selective serotonin reuptake inhibitor SNRI, selective norepinephrine reuptake inhibitor

## Data Availability

The datasets analyzed for the current study will be made available on an individual basis upon reasonable request to the corresponding author.
